# Age-Period-Cohort Projections of Ischaemic Heart Disease Mortality by Socio-Economic Position in a Rapidly Transitioning Chinese Population

**DOI:** 10.1371/journal.pone.0061495

**Published:** 2013-04-11

**Authors:** Irene O. L. Wong, Benjamin J. Cowling, Gabriel M. Leung, C. Mary Schooling

**Affiliations:** 1 Lifestyle and Life Course Epidemiology Group, School of Public Health, Li Ka Shing Faculty of Medicine, The University of Hong Kong, Hong Kong SAR, China; 2 CUNY School of Public Health at Hunter College, New York, United States of America; Cardiff University, United Kingdom

## Abstract

**Background:**

With economic development and population aging, ischaemic heart disease (IHD) is becoming a leading cause of mortality with widening inequalities in China. To forewarn the trends in China we projected IHD trends in the most economically developed part of China, i.e., Hong Kong.

**Methods:**

Based on sex-specific IHD mortality rates from 1976 to 2005, we projected mortality rates by neighborhood-level socio-economic position (i.e., low- or high-income groups) to 2020 in Hong Kong using Poisson age-period-cohort models with autoregressive priors.

**Results:**

In the low-income group, age-standardized IHD mortality rates among women declined from 33.3 deaths in 1976–1980 to 19.7 per 100,000 in 2016–2020 (from 55.5 deaths to 34.2 per 100,000 among men). The rates in the high-income group were initially higher in both sexes, particularly among men, but this had reversed by the end of the study periods. The rates declined faster for the high-income group than for the low-income group in both sexes. The rates were projected to decline faster in the high-income group, such that by the end of the projection period the high-income group would have lower IHD mortality rates, particularly for women. Birth cohort effects varied with sex, with a marked upturn in IHD mortality around 1945, i.e., for the first generation of men to grow up in a more economically developed environment. There was no such upturn in women. Birth cohort effects were the main drivers of change in IHD mortality rates.

**Conclusion:**

IHD mortality rates are declining in Hong Kong and are projected to continue to do so, even taking into account greater vulnerability for the first generation of men born into a more developed environment. At the same time social disparities in IHD have reversed and are widening, partly as a result of a cohort effect, with corresponding implications for prevention.

## Introduction

**Figure 1:**
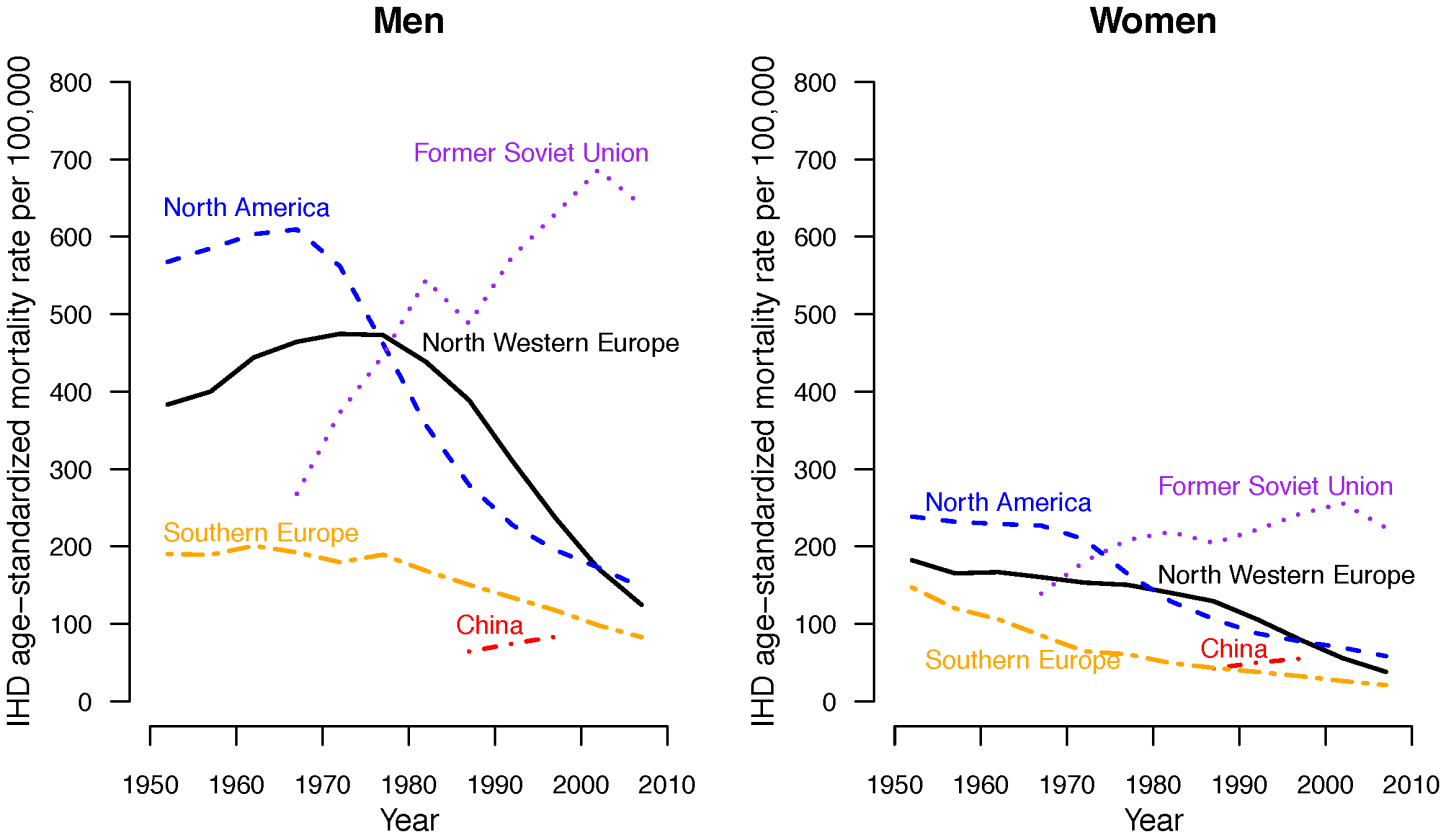
Age-standardized IHD mortality rates per 100,000 male/female population in North Western Europe, North American, Former Soviet Union, China and Southern Europe from 1950 to 2010. (Notes: Data for the Former Soviet Union and China are limited due to data availability; data are retrieved from the WHO mortality database[45] and publications [46] and standardized to the WHO world standard population).

Ischemic heart disease (IHD) is one of the leading causes of death in both China [1]. With economic development and the epidemiologic transition, deaths from IHD in China are expected to approximately double between 1990 to 2020, with a greater impact in men [2], [3]. Epidemics of IHD among men with economic development have varied immensely from very marked epidemics in North American, North Western Europe and more recently in the former Soviet Union to much less marked epidemics in Southern Europe (Figure 1). The Seven Countries Study found that differences in dietary patterns might explain different IHD rates between those regions studied. Reasons for these differences have never been fully elucidated, but instead have been attributed to unexplained differences in ‘baseline’ risk for which risk prediction models are recalibrated. Consequently it is not known how the epidemic of IHD may develop as one fifth of the world's population in China proceeds rapidly through a period of immense economic transition. The Chinese population of Hong Kong has already experienced over a lifetime the economic transition underway in China, and thus may act as a sentinel for changes currently taking place in China.

In several ways the epidemic of IHD in Hong Kong shares cardinal features with those observed elsewhere. During the IHD epidemic, in western populations, IHD may reverse social patterning from being positively associated with socio-economic position to being negatively associated with socio-economic position, particularly among men [4]. A similar reversal has recently been observed in Hong Kong [5]. The Ni Hon San seminal study in the 1970s of Japanese men living in Japan or in the United States showed that growing up in a more developed environment was associated with a higher risk of IHD[6], [7]. We have similarly shown that the first generation of men, but not women, to grow up in Hong Kong, following mass migration in the mid 20^th^ century from pre-industrial China to economically developing Hong Kong, had a relatively higher risk of death from IHD than previous generations [8]. To clarify the effects of economic transition on the IHD epidemic in a rapidly developed Chinese population, we used a model that identifies trends due to early life influences, contemporary influences and aging to make sex-specific projections of IHD mortality in Hong Kong taking into account socio-economic position. In the model, we decomposed IHD mortality over the last 30 years into the effects of birth cohort, calendar time period and chronological age at death [9] for men and women by socio-economic position (i.e., low or high income). We then projected the effect of each of these components separately in the Hong Kong population to give sex-specific estimates of IHD mortality rates until 2020 by income group.

## Methods

### Data

#### Mortality rates and population

We obtained sex-specific IHD deaths (IHD defined according to the International Classification of Diseases (ICD) codes as ICD-8 410-414, ICD-9 410-414 and ICD-10 I20-I25) and mid-year population figures by age group at the neighborhood or Tertiary Planning Unit (TPU) level from the Hong Kong Death Registry and the Government Census and Statistics Department (C&SD) in Hong Kong for the years 1976–2006, respectively. In Hong Kong, the entire population is divided into 287 standard TPUs by the Government Planning Department. TPUs typically represent geographical areas bounded by roads, railway lines, coastlines, contours, waterways, lot boundaries or zoning boundaries of town plans. TPUs' boundaries are regularly updated to reflect population dynamics, although some are sparsely populated. Sparsely populated TPUs are merged with geographically near-by TPUs by the C&SD before the data is released to ensure anonymity of the inhabitants. In the analyses, the original set of 287 TPUs was collapsed into approximately 172 TPUs according to standard methods of the C&SD, due to small cell sizes in some of the less populated areas.

The Hong Kong Death Registry is the population-based registry of deaths for the Hong Kong population. We included all IHD deaths registered during the period of observation. Given that IHD mortality is rare for children and teenagers, we grouped the deaths and population data into ten 5-year age groups from 30 to 34 years to 75 or above, and six 5-year calendar time periods from 1976–1980 to 2001–2005, respectively. This classification resulted in 15 birth cohorts centered at 5-year intervals beginning in the 1901.

#### Socio-economic position

Measures of socio-economic position, such as education or income, are not available on death certificates. We obtained monthly median household income data at the neighborhood (TPU) level from the Population Census at 1976, 1981, 1986, 1991, 1996, 2001 and 2006 in Hong Kong. We classified deaths and the population according to the socio-economic position of their residential neighborhood (TPU) assessed from median household income per capita. TPUs with median household income per capita below the median for all TPUs were classified as low-income. TPUs with median household income per capita equal to or above the median for all TPUs were classified as high income. We classified deaths as low- or high-income based on the neighborhood median household income per capita of their TPU of residence at the time of death. We similarly classified the population as low- or high-income based on the neighborhood median household income per capita of their TPU of residence.

#### Statistical analyses

Age-adjusted mortality rates were calculated by direct standardization according to the World Standard Population [10] and expressed per 100,000 people by sex and income group, since this is the reference population currently adopted by the Hong Kong Death Registry.

We modeled IHD mortality using an age-period-cohort model [11], [12] which decomposed mortality rates over time by chronological age, calendar period and birth cohort. We used the second and penultimate periods and the central birth cohort as reference categories, in order to generate identifiable estimates for birth cohort and period effects respectively. We used Bayesian inference to estimate the model parameters [13], and the fitted model was used to project future mortality rates in 3 further 5-year periods up to 2016–2020.

For the age, period and cohort effects we specified second-order Gaussian autoregressive priors in the forward direction [13]. These priors specified that the initial expected value of each effect is based on an extrapolation from its 2 immediate predecessors. We extrapolated 3 additional period and cohort effects to give projections of future deaths. We estimated the model parameters using Markov Chain Monte Carlo (MCMC) simulations with 5 concurrent chains started at different initial values since comparison of multiple chains allows us to discern convergence. We used the criteria R-hat to monitor convergence [14]. On the basis of the values of R-hat, we decided how many of the initial samples to discard as a burn-in period, and then took a further 40,000 samples from the posterior distributions. The parameter estimates and derived rates were summarized in terms of posterior means and 95% credible intervals. The model goodness-of-fit was measured by the posterior mean deviance D [15]. To compare models, the deviance information criterion (DIC) was calculated, which adjusted the posterior mean deviance for the number of parameters in the model [15]. A smaller DIC implies a better fit.

To examine whether the results differed by socio-economic position, we investigated any potential differences in birth cohort, calendar period or chronological age effects for men or women by stratifying by socio-economic position (i.e., income group). We used the resulting models to project age-standardized IHD mortality rates by sex and by income group until 2020. We also applied join point regression to evaluate whether or not there has been any change in the trends in cohort and period effects over time and when change occurs (whenever applicable) [16]. The best join point model selection method was based on Bayesian Information Criterion BIC where the best model was selected with the smallest BIC [16].

All analyses were implemented using R version 2.10.1 (R Foundation for Statistical Computing, Vienna, Austria), WinBUGS version 1.4 [17] and Join point regression software program version 4.0.1 from the surveillance research program of the US National Cancer Institute.

## Results

**Figure 2:**
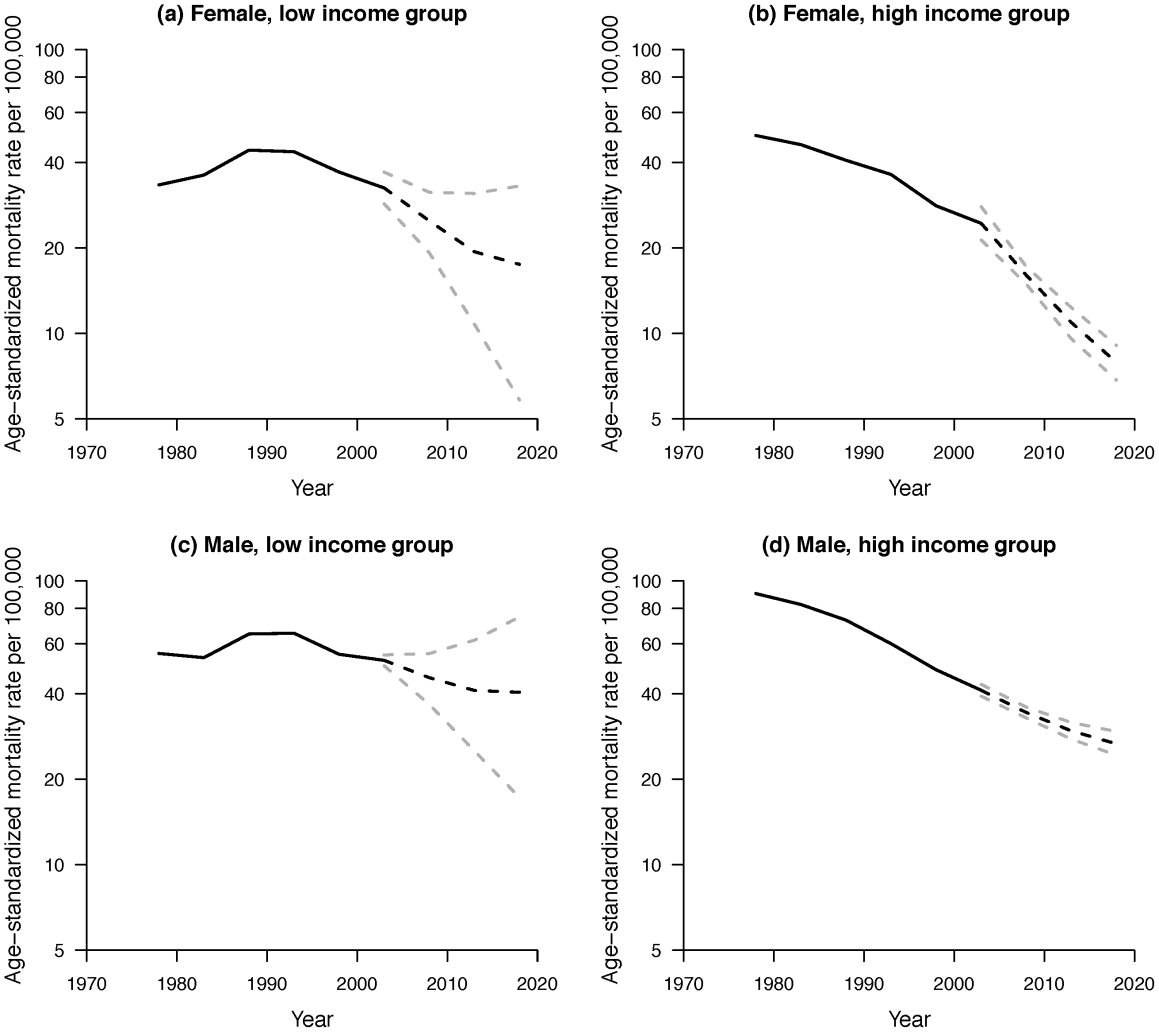
Annual age-standardized IHD mortality rates in Hong Kong from 1976 to 2005 (solid lines) and projected mortality rates to 2020 (dotted lines) with 95% credible intervals (gray dotted lines) for (a) Female, low income group, and (b) Female, high income group, (c) Male, low income group and (d) Male, high income group.

Figure 2 shows the age-standardized mortality rates from IHD per 100000 women and men from 1976 to 2005 and the projected IHD mortality rates from 2006–2010 to 2015–2020 by income group. At the start of the period considered the IHD mortality rate was higher for the high income group than for the low income group, particularly among men, but this had reversed by the end of the period. The IHD mortality rate declined faster for the high income group than for the low income group. Our model projected IHD mortality rates continuing to decline faster in the high income group, such that by the end of the projection period the high income group had much lower IHD mortality rates, particularly for women.

**Figure 3:**
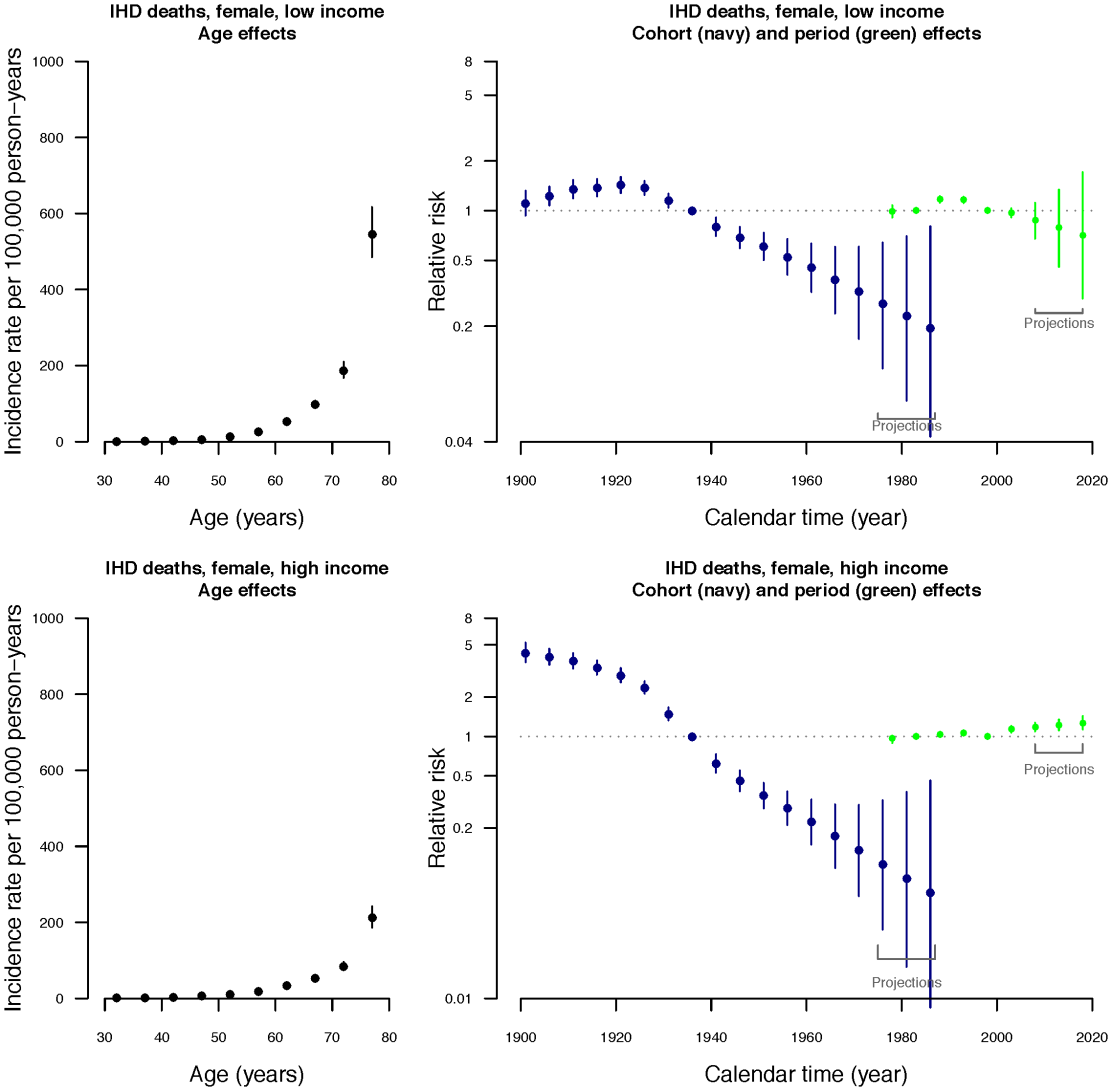
Parameter estimates of age, period and cohort effects from the age-period-cohort model (DICs = 453.604 for low income group; 443.02 for high income group). Left hand panel: Estimated age-specific annual *female* mortality rates due to IHD in 5-year age groups in Hong Kong with 95% credible intervals. Right hand panel: Estimated relative risks for 10-year birth cohorts (beginning in the calendar year 1899) and 5-year calendar periods (beginning from 1976) with 95% credible intervals, including projected cohort effects for birth cohorts centered on the years 1978, 1983 and 1988 and projected period effects for periods centered on the years 2008, 2013, 2018.

**Figure 4:**
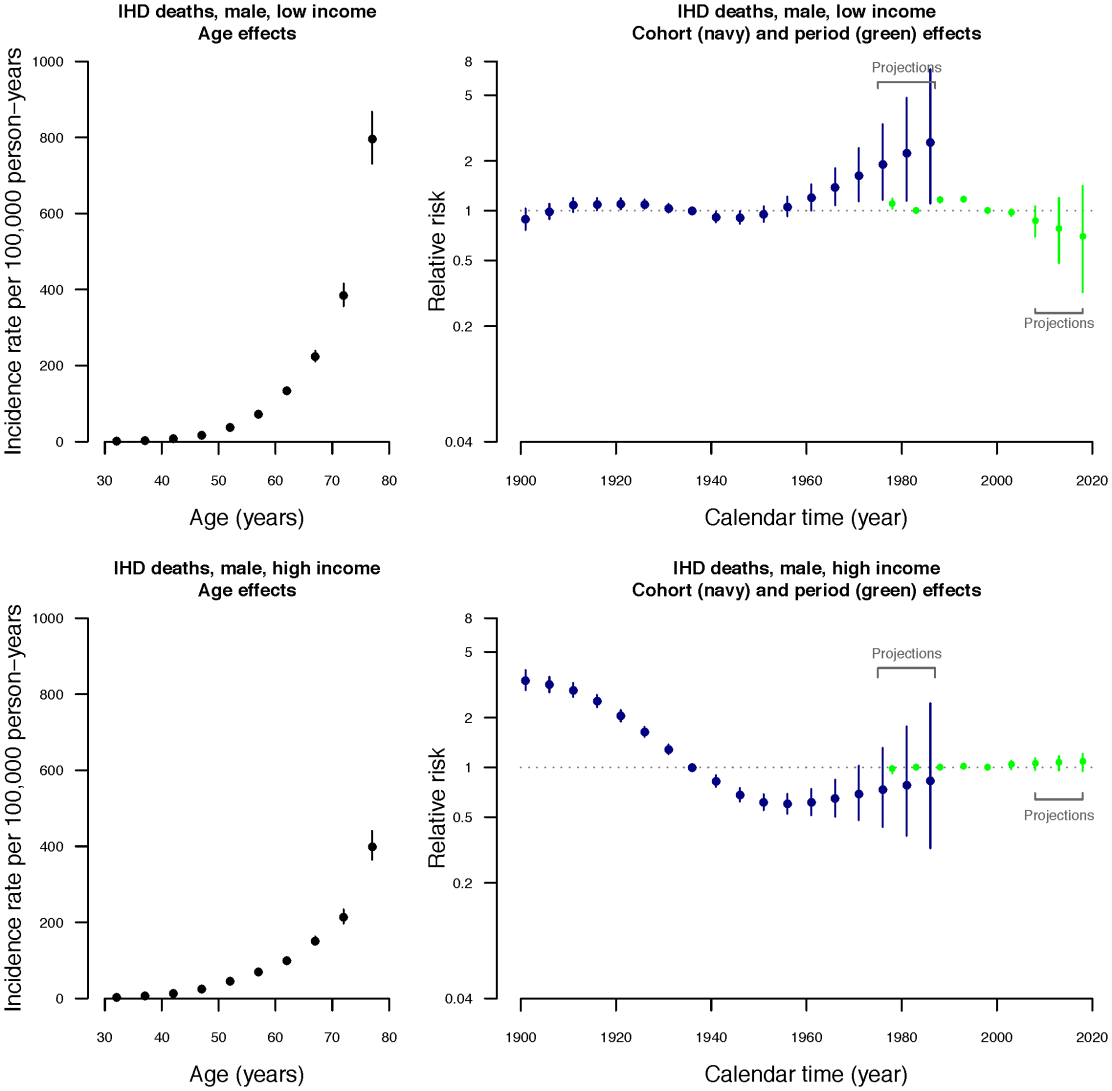
Parameter estimates of age, period and cohort effects from the age-period-cohort model (DICs = 526.797 for low income group; 569.726 for high income group). Left hand panel: Estimated age-specific annual *male* mortality rates due to IHD in 5-year age groups in Hong Kong with 95% credible intervals. Right hand panel: Estimated relative risks for 10-year birth cohorts (beginning in the calendar year 1899) and 5-year calendar periods (beginning from 1976) with 95% credible intervals, including projected cohort effects for birth cohorts centered on the years 1978, 1983 and 1988 and projected period effects for periods centered on the years 2008, 2013, 2018.

Age, period and cohort contributed to all models, although the contribution of period was relatively minor for high-income men and women (Table S1). The estimated parameter values of age, period and cohort components with projections are shown in Figures 3 and 4. Because of the known identifiability problem of APC models, where there is inherent linear independence between the three component effects (i.e., birth cohort  =  period of deaths – age at death), only second-order changes (i.e., infection points or changes in slopes) are interpretable [9], [18], [19], [20]. The left panel of Figures 3 and 4 shows the fitted age-specific mortality rates per 100000 women and men respectively by income group. Among men and women, mortality rates increased with age in both income groups, with an acceleration at the age of approximately 50 years. Age-specific mortality rates were higher for the low income group than the high income group, particularly at older ages.

Relative risks were estimated for the fifteen 10-year birth cohorts beginning in the calendar year 1901 and the six 5-year time period from 1976 to 2005 (right panel of Figures 3 and 4). Cohort effects dominated, and inflection points can be clearly identified, while second-order changes in period effects were less apparent. Regarding the cohort effects, a downturn occurred for cohorts born in the 1920–25 for both high and low income women, followed by a very slight upturn for high income women around 1940–45. In contrast, the cohort effects for men were quite different. The downturn appeared to be slightly earlier for men than for women, i.e., around about 1910–15, whilst for both high and low income men a marked upturn occurred around 1945–50, which was more obvious for low income men. In the join point analyses, the join-points that we identified were consistent with the inflection points described above.

Based on the APC analyses, period effects were more evident for the low-income than the high-income group, and a downturn occurred in the early 1990s followed by an upturn in the late 1990s. Similar results were found in join point analyses for high income men and women, however, changes in linear trends in period effects took place at c. 1988 and c. 1998 for low income women, and c. 1993 for low income men.

## Discussion

Economic transition over a compressed time frame in a Chinese population resulted in reducing IHD mortality rates for both men and women that are projected to continue with widening socio-economic disparities, mainly due to a cohort effect, although there was a relatively higher risk for the first generation of men to grow up in improved living conditions (Figure 4). We did not find any clear evidence of an emerging epidemic of IHD among men similar to those which occurred in North America and Northern Europe with economic development in the mid 20^th^ century [21].

Our findings might have implications on ischemic stroke trends, which usually followed the pattern of IHD trends. At the same time, hemorrhagic stroke may decline with economic development, as has been observed in Europe [22] and possibly in mainland China [23].

Generally our findings are similar to those seen elsewhere in terms of changing social disparities in IHD with economic development [4]. Our analysis adds by showing this disparity is driven by age and cohort effects as well as by current experiences. Few other analyses have examined disparities in terms of cohort effects, however a study from China also showed that differences by cohort contributed to increasing inequalities [24].

The period effects were more marked for the low income group suggesting that the disadvantaged are more sensitive to population wide changes, both positive and negative. This seems intuitive, however few other studies have considered social disparities by age, period and cohort. The establishment of the Hospital Authority, which is the central authority managing about 90% of total bed-days in Hong Kong by 44 public hospitals, in the early 1990s coincides with a more marked downturn for the low income group, perhaps because they obtained greater benefit from any associated improvements in public services. Similarly, the Asian financial crisis in 1997 also coincides with a more marked upturn for the low income group.

The low income group was more vulnerable to IHD at younger ages. This may suggest faster aging, higher risk factor exposures, or higher adverse environmental exposures in the low income group.

Our study is similar to many others in showing that the decline in IHD is mainly due to a cohort effect [25], [26], [27], [28], [29], [30], although there are exceptions, for example in Australia [31] and New Zealand [32]. However, almost all these studies relate to settings (Figure 1) where an epidemic of ischemic heart disease occurred in the mid 20^th^ century, and thus do not elucidate why the original epidemic occurred [33]. Our analysis suggests that an unidentified factor associated with growing up in a more developed environment can contribute to an epidemic of IHD among men. However, in our particular setting it does not have a very large effect. We have previously suggested that up-regulation of the axis controlling growth with economic development underlies the corresponding changes in patterns of disease, with specifically up-regulation of the gonadotropic axis underlying the epidemic of IHD among men [34]. Currently men in Hong Kong have lower peak androgen levels than Caucasian men [35], so it may take a few more generations before an epidemic of IHD emerges among Chinese men in Hong Kong. Similarly among women in Hong Kong an increase in risk of another hormone related disease, i.e., breast cancer, occurred for the first generation of women to grow up in Hong Kong [36]. However, breast cancer rates in Hong Kong remain lower than those in long-term developed western populations, we suggest, because there have not been sufficient generations of economic development for the drivers of breast cancer to build up to the levels seen in the west [36]. Hence the possibility remains that an epidemic among men of IHD may emerge in Hong Kong and by implication in China after additional generations of economic development.

It may seem counter-intuitive that IHD rates are projected to decrease in Hong Kong when some IHD risk factors are high in Hong Kong, such as diabetes[37], or likely to increase as the population becomes increasingly sedentary and westernized. However, it is well-known that traditional IHD risk factors do not always predict well in non-western populations. For example, the Framingham score very substantially over-predicts absolute risk of IHD events in China [38], [39]. Again, reasons for this over-prediction are not well understood. However, it is becoming increasingly clear that the Framingham score is based on risk factors, which may not be causal. For example the causal role of HDL-cholesterol in IHD has been challenged in several recent studies of different designs [40], [41], [42], suggesting HDL-cholesterol predicts well as an indicator of some other underlying factor. Notably, androgens generate unfavourable levels of HDL-cholesterol [43], however there could be many other potential factors which lower HDL-cholesterol and cause IHD. Nevertheless, the items in the Framingham score may simply correlate better with the underlying causal factors in some populations rather others.

Several caveats bear mention. First, we need to be cautious in interpreting the APC model, as we can only speculate about the etiologies of the observed changes. In fact, we examined the model from a practical perspective to ensure it is grounded in reality. We also examined whether any aspects of the model could be the artefactual results of data changes, such as coding changes or other specific events that have taken place during the period. Second, we used community-level income at the time of death relative to contemporary population median income at the community-level to classify relative socio-economic position, which provides a relative classification with a different cut point each year rather than a classification relative to the same level of neighborhood income throughout the 30 year period. However, given the level of economic growth during the 30 year period classification using one (cost of living adjusted) cut point for the entire period would have resulted in most of the population in the early years being classified as low income and most of the population in the later years being classified as high income. Moreover, it is not clear that community-level income would reflect average individual socioeconomic position. We also did not include other community-level factors into the analyses. However, we speculate that there is no significant difference of community characteristics in terms of access to fresh food, physical activity facilities and quality of health care, given Hong Kong has universal population coverage in health care services in the public sector (but subject to co-payments at the point-of care and capacity of the services), with high population density throughout the territory, excellent travel infrastructure and connectivity and a high degree of social integration. Third, for older people income of their residential location at the time of death may not reflect their lifelong income position in the social hierarchy. Fourth, observed trends might be also driven by changes in the well-known risk factors such as diet, physical activity, and tobacco smoking. However, the trends over the same period could not be analyzed due to lack of reliable past data. Lastly, the “healthy” migrant effect may have masked differences in mortality between the high and low income groups. However, almost the entire adult population of Hong Kong is first or second generation migrants [44].

## Conclusions

Our study suggests that IHD mortality rates will continue to fall in Hong Kong among both men and women. Our study does not suggest that an epidemic of IHD among men in China is imminent, despite the epidemics which have occurred in other settings with economic development, perhaps because of insufficient generations of economic development. Thus we cannot rule out the possibility that an epidemic of IHD among men in Hong Kong and similar population, such as in China, may occur later in the 21^st^ century. Our study also suggests widening inequality in IHD mortality will occur in Hong Kong and similar settings, such as China, which may be associated with a cohort effect, meaning that programs to reverse such inequality will need to extend over the life course and may take years to bear fruit. As such, attention should be focused on identifying and ameliorating the factors which generate inequalities throughout life.

## Supporting Information

Table S1 Deviance information criterion (DIC) values for different combinations for age, period and cohort models for deaths due to ischemic heart disease in Hong Kong
(DOCX)
