# Supplementary material for: Age-Period-Cohort Projections of Ischaemic Heart Disease Mortality by Socio-Economic Position in a Rapidly Transitioning Chinese Population
Source: PLoS One. 2013 Apr 11;8(4):e61495. doi: 10.1371/journal.pone.0061495 (PMC3623955; doi:10.1371/journal.pone.0061495)
Supplement: Table S1 — Deviance information criterion (DIC) values for different combinations for age, period and cohort models for deaths due to ischemic heart disease in Hong Kong (DOCX) [file pone.0061495.s001.doc]

# Appendix Table 1. Deviance information criterion (DIC) values for different combinations for age, period and cohort models for deaths due to ischemic heart disease in Hong Kong

| Model | Components | DIC | |
| --- | --- | --- | --- |
|  |  | High-income | Low-income |
| Female |  |  |  |
| 1 | Age | 1892 | 884 |
| 2 | Period | 3553830 | 15246200 |
| 3 | Cohort | 15365600 | 4114350 |
| 4 | Age, period | 1151 | 642 |
| 5 | Age, cohort | 451 | 518 |
| 6 | Age, period, cohort (Full model) | 441 | 454 |
| Male |  |  |  |
| 1 | Age | 2600 | 772 |
| 2 | Period | 14930600 | 15769400 |
| 3 | Cohort | 3966720 | 4114350 |
| 4 | Age, period | 1293 | 590 |
| 5 | Age, cohort | 570 | 616 |
| 6 | Age, period, cohort (Full model) | 570 | 527 |
